# New surgical approach for late complications from spinal cord injury

**DOI:** 10.1186/1471-2482-6-12

**Published:** 2006-10-23

**Authors:** Antonio J Reis

**Affiliations:** 1 Clínica Santa Catarina, 9000-045, Funchal, Madeira – Portugal, And Hospital Sant'Ana, Parede, Portugal

## Abstract

**Background:**

The most frequent late complications in spinal cord injury result from arachnoiditis and consequent alterations in dynamics of cerebrospinal fluid flow. A surgical procedure carried out on patients with these alterations, resolved the various pathologies more efficiently in all cases.

**Methods:**

From October 2000 to March 2006, 23 patients were selected for surgery: three showed signs of syringomyelia, three presented with microcystic lesions, three presented with arachnoid cysts in different locations but always confluent to the scar area, and 14 showed evidence of tethered cords. The surgery consisted of laminectomy at four levels, followed by dural opening in order to remove all the arachnoiditis at the level of the scar and to remove the altered arachnoid and its cysts, at least at two levels above and below the lesion. The dentate ligaments were cut at all exposed levels.

**Results:**

The patients had no postoperative problems and not only retained all neurological functions but also showed neurological recovery. According to the motor and sensory scale of the American Spinal Injury Association, the recoveries were motor 20.6% (P < 0.001), touch 15.6% ((P < 0.001) and pinprick 14.4% (P < 0.001). These patients showed no signs of relapse at 4–66 month follow-up.

**Conclusion:**

This alternative surgery resolved the pathologies provoking neurological deterioration by releasing the complete spinal cord at the level of the scar and the levels above and below it. It thus avoids myelotomies and the use of shunts and stents, which have a high long-term failure rate and consequent relapses. Nevertheless, this surgical procedure allows patients the chance to opt for any further treatment that may evolve in the future.

## Background

Arachnoiditis is one of most important factors in late complications of spinal cord injury patients. It leads to intra-medullar lesions such as syringomyelia cysts and microcysts, or extra-medullar lesions such as tethered cords and arachnoid cysts. All these pathologies were well described and differ in their clinical manifestations and in the forms in which they could be diagnosed. Abnormalities common to all the pathologies include arachnoiditis and altered cerebrospinal fluid (CSF) dynamics, which create the conditions for various pathologies, to appear in different ways in patients [1-11]. Sometimes these clinical alterations appear very slowly and insidiously, and could not be perceived by the patient. However, only 1/4 of all patients show clinical evidence of neurological deterioration, which can be revealed by magnetic resonance imaging (MRI) or conventional myelographic computed tomography.

For each of the situations referred above, various surgical procedures are available as described in assorted publications together with results [12,13]. However, the underlying conditions can lead to relapse, as happens frequently in a reoperation for syringomyelia, in which it is common to find the shunt dislocated or obstructed by the intense adhesive reaction of the arachnoid. Notwithstanding cases of non-traumatic arachnoid cysts, neurosurgeons never limit surgery to the CSF input to the subarachnoid space below the cyst, but remove the arachnoid completely along with its adhesions that produced the cyst. Nevertheless, arachnoids cysts with traumatic origins must be treated in the same way [14,5].

Many materials are currently available for fixing and stabilizing the spinal traumatic canal in patients who have instability after acute spinal cord injury (SCI). When such a patient who has been operated for stabilization needs a second operation, it has been verified that complete re-alignment of the canal is rarely obtained; it is incomplete in 72% of this group of patients [16,17], and [18]. Therefore, an unknown percentage of patients will show another associated traumatic myelopathy that will lead to a ring-shaped dural fibrosis at the level of compression. These consequences cause compression of all sub-dural structures and provoke changes in the dynamics of CSF flow.

After previous surgical techniques had been evaluated, a new approach was proposed. The first two patients operated within this series presented with syringomyelia. Instead of conventional surgery, the medulla was completely freed of the fibrotic webs, bridges, and attachments that surrounded it. The dentate ligaments were cut to access the ventral face of the cord. With the medulla free and CSF circulation probably close to normal, it was decided to avoid myelotomy for shunting since there was no arachnoidal tissue to fix the shunt. The symptoms that motivated the surgery disappeared, and in control examinations, it was possible to evaluate the regression of the syrinx cysts.

## Methods

Surgical operations took place at the Orthopaedic Hospital of Sant'Ana, Parede, after the complete approval of the Clinical Director and the President of the Scientific Committee of the Hospital, who is also the Orthopaedic Director. Before the procedure, full consent from the patients and assent from their relatives were obtained. Phase one of this work was initiated in October 2000 and completed in March 2003. After complete clinical data had been collected for 14 patients, it was decided to recruit another nine patients to the study; this was done between the middle of 2004 and March 2006.

The first two patients operated on had post-traumatic syringomyelia and the others were as follows: three patients with multiple microcystic lesions, one with a syrinx cyst, 3 with arachnoid cysts and 14 with tethered cords. Their initial symptoms were an increase in spasticity, sensory and motor deterioration and an increase of local and radicular pain.

These patients were selected according to the following three criteria: first, all patients in MRI and CT and 14 in CT myelography who presented with anatomical continuity of the cord with one single level of lesion and evidence of the main pathology. Second, patients who had been operated for realignment and stabilization of the spine at some time after the acute spinal cord injury. Third, those who continued physiotherapy post accident. For the second cohort of patients (2004–2006) recruited after experience had been gained from the first cohort, it was decided to add an extra inclusion criterion: a different rehabilitation program for a period of at least 6 months before surgery [19,20]. In addition to imaging studies, all patients underwent a complete medical and neurological examination to determine correctly their actual medical and neurological condition and its evolution after the accident. All clinical records and images, studies undertaken at the time of the accident and those undertaken thereafter were evaluated before any surgical decision was made. The patients selected included 4 with different cervical injuries and 19 with thoracolumbar injuries, having the following different kinds of lesions: five showed complete lesions (21.7%), eight were motor complete and sensory incomplete (34.7%), and nine showed both motor and sensory incompleteness (43.4%). Three were women (13.1%) and 20 were men (89.9%) and the age range was from 8 to 54 years (mean 29.3 years); intervals between the SCI and the operation ranged from 6 months to 20 years (mean 5.1 years); see Table 1.

**Table 1:** Clinical data of selected patients.

Patients	Sex/age	Level of injury	Neurological outcome	Time between acute injury and surgery	Diagnosis
1	Ma-22	T-3	Asia-A	4 years	syrinx
2	Ma-28	C-6	Asia-A	9 years	syrinx
3	Ma-49	T-4	Asia-C	19 years	Tethered cord
4	Ma-26	L-2	Asia-B	9 years	Tethered cord
5	Ma-32	T-12	Asia-C	5 years	Tethered cord
6	Ma-30	T-11	Asia-B	4 years	Microcysts
7	Fe-23	T12	Asia-B	8 months	Tethered cord
8	Ma-26	T-8	Asia-B	2 years	microcysts
9	Fe-11	T-11	Asia-A	11 months	Tethered cord
10	Ma-23	C-5	Asia-B	3 years	Tethered cord
11	Ma-27	T10	Asia-B	10 years	syrinx
12	Ma-28	T5	Asia-B	10 years	microcysts
13	Fe-21	T-10	Asia-C	26 months	Arachnoid cyst
14	Ma-37	T-12	Asia-C	2 years	Tethered cord
15	Ma-30	T-12	Asia-C	3 years	Tethered cord
16	Ma-30	T-5	Asia-C	5 years	Arachnoid cyst
17	Ma-34	L-1	Asia-C	5 years	Tethered cord
18	Ma-22	T-6	Asia-A	15 months	Tethered cord
19	Ma-37	T-6	Asia-C	8 months	Tethered cord
20	Ma-21	C-7	Asia-B	11 months	Tethered cord
21	Ma-8	T-6	Asia-A	6 months	Tethered cord
22	Ma-46	C-5	Asia-C	20 years	Arachnoid cyst
23	Ma-54	T12	Asia-C	2 years	Tethered cord

Patients 2, 7, and 10 were out of the follow up.

With different diagnosis, they have in common the arachnoiditis and altered cerebrospinal fluid flow dynamics.

Neurological follow up was conducted every month over the first six months and then evaluations continued every 3 months until the end of the first year. Nowadays all patients undergo neurological evaluations every six months. It must be emphasized that the greatest neurological recovery occurred in the first months after surgery, as shown in Table 2. The American Spinal Injury Association (ASIA) score scale results in the first year were: Motor-13 points, Touch-11 points and Pinprick-8 points, which represented more than 50% of the latest follow up. A control MRI was obtained at the end of the first and third years.

**Table 2:** Clinical evolution of patients

**ASIA motor and sensory recovery**												
Patient/Lesion	**Initial**			**Follow up (up to one year)**			**Last Follow up**			**Results (%)**		
	
	Motor	Touch	Pinprick	Motor	Touch	Pinprick	Motor	Touch	Pinprick	Motor	Touch	Pinprick

1-comp	50	36	36	64	57	62	72	71	77	22,0%	31,3%	36,6%
2-inc	60	46	46	73	61	61	80	72	70	20,0%	23,2%	21,4%
3-inc	50	84	84	70	98	98	79	100	100	29,0%	14,3%	14,3%
4-inc	61	72	70	79	79	72	87	86	72	26,0%	12,5%	1,8%
5-inc	50	68	68	60	73	73	66	75	76	16,0%	6,3%	7,1%
6-inc	50	54	52	65	63	60	72	68	66	22,0%	12,5%	12,5%
7-comp	50	68	68	69	70	70	78	70	68	28,0%	1,8%	0,0%
8-inc	62	52	56	75	66	71	80	75	68	18,0%	20,5%	10,7%
9-inc	50	58	52	61	62	60	68	62	62	18,0%	3,6%	8,9%
10-inc	50	72	54	64	77	72	71	80	68	21,0%	7,1%	12,5%
11-inc	56	74	72	66	82	77	73	89	80	17,0%	13,4%	7,1%
12-inc	66	80	80	73	85	83	78	89	86	12,0%	8,0%	5,4%
13-inc	56	58	56	68	65	61	76	70	68	20,0%	10,7%	10,7%
14-inc	62	71	70	77	84	83	84	90	88	22,0%	17,0%	16,1%
15-comp	50	56	56	67	71	67	78	80	77	28,0%	21,4%	18,8%
16-inc	26	28	26	47	55	50	58	70	66	32,0%	37,5%	35,7%
17-inc	56	70	70	65	77	77	72	82	82	16,0%	10,7%	10,7%
18-comp	26	24	24	35	50	50	40	68	62	14,0%	39,3%	33,9%
19-inc	56	84	82	68	87	85	72	90	90	16,0%	5,4%	7,1%
20-inc	50	48	46	59	62	61	64	66	64	14,0%	16,1%	16,1%

**Mean Values**	52	60	58	65	71	66	72	78	75	20,6%	15,6%	14,4%

Initial neurological evaluation and recovery in score points and percentage

## Surgical technique

This consisted of a laminectomy at four levels with the scar lesion in the centre of the surgical field. The dura mater was opened to make all the scar tissue visible, and the extremities were easily identified by the presence of the arachnoid cysts. When necessary, the laminectomy was expanded one level further above or below (six patients), until normal arachnoid tissue and spinal cord appeared. When the dura was opened, the first step was the opening of the arachnoid cysts situated rostral and caudal to the lesion in order to find the layer between the thickened arachnoid and the spinal cord. This layer was found on both sides, above and below. With arachnoiditis tissue fully attached to the dura, some traction could be applied to this thickened tissue, so that dissection could be started on all the dorsal face of the cord between the extremities of lesion above and below to the scar direction. At this point, of the major compression, the arachnoiditis tissue was removed fragment by fragment in order to avoid vascular or especially spinal cord lesions, although some fragments were left behind. The same procedure was performed on both lateral faces of the cord, moving it away from the roots. In one patient, both T-5 roots were cut to achieve a good dissection. The dentate ligaments were cut at all exposed levels. After removal of the fibrotic tissue from the cord posteriorly and laterally, the remainder, which was fully attached to dura mater, was removed. At this stage, it was possible to see that the spinal cord remained in very high tension owing to the remaining ventral adhesions. An anterior dissection was made as far from the mid-line as possible, then the spinal cord was pushed away to the mid-line from both sides with small and light movements in order to release it completely. Long tractions to the roots were avoided. When the spinal cord was finally released on its ventral face, the visible tension disappeared immediately and it was possible to move the cord from both sides. In some cases, to ensure that the spinal cord was completely released, a cotton Pattie was introduced at one side below the cord and removed from the other side. Any bone or disk fragments, or even small angulations of the canal, were removed or corrected, through a small incision parallel to the cord (five patients). This made it possible to re-establish the posterior wall of the fractured vertebra. Once the dura and spinal cord were completely free from adhesions, the dura mater was closed without augmentation duroplasty.

In an 8-year old patient, a thoracic laminotomy was performed to avoid further instability. In all the patients, the material used to stabilise the spinal canal was fully maintained.

## Results

Three patients abandoned the "follow-up" (patients 2,7,10 in Table 1). The remaining 20 had "follow-ups" ranging from 4 months to 66 months. There were no postoperative complications or temporary neurological deficits in any patient after surgery, and all patients with incomplete lesions maintained neurological functions below the injury site.

The results were evaluated from two points of view. The first was evaluation with MRI and computed tomography in the first and third years after surgery. The control MRI was compared with the previous examination. It was found that all subdural and extra-medullar lesions disappeared and CSF circulation was close to normal. The intra-medullar lesion, which presented as an unenhanced sagittal T1 weighted image of decreased signal intensity, was reduced in respect of sagittal size, but was present at the site of major compression. In T2 weighted images, a reduction of the medullar areas of hyperintensity was seen around the area of major compression. In the 3 cases of syrinx there was also complete regression of the cysts; in only 2 patients did some evidence of the location of the cysts remain. After three years, one of the patients showed spinal cord expansion or better-defined spinal cord walls in MRI images when compared with previous examinations.

The second criterion of evaluation was totally clinical, not only was neurological deterioration eradicated, but some neurological functions appeared below the level of the injury. Although neurological recovery had never been the reason for these operations, all patients subjected to this procedure showed some recovery of motor and sensory functions. In Table 1, the diagnoses show different pathologies with the same origins, arachnoiditis and altered CSF circulation, and all of them were treated by the same spinal cord untethering procedure. The 14 patients with tethered cord syndrome have an average follow up of 48.6 months and to date have suffered no relapses, which indicates a good result.

Altogether, the complete group of 20 patients with follow-up from four to 66 months continues to present good neurological recovery. According to the ASIA motor and sensory score scale the recovery averages were: Motor – 22 points, Touch – 18 points and Pinprick – 17 points (Table 2). In the same table, the percentage [see Additional file 2] presented was: Motor 20.6%, Touch 15.6% and Pinprick 14.4%. The Wilcoxon signed ranks test [Additional file 1] confirms P < 0.001 for all modalities.

## Discussion

There are surgical solutions for the various pathologies, that may appear (with some delay) after a SCI, and they are published with results in many series for each pathology. However, some percentage of relapses is normal and these are intimately linked to obstructions and dislocations of the CSF drainage system. Some authors have performed laminectomies in the area of the injury to effect neurological improvement. However, the results have not been statistically significant so this technique is rarely used. Nevertheless, some patients showed a measure of recovery, probably due to decompression of the intra-dural structures and some alteration in CSF dynamics [21,22]. It becomes evident, experimentally and in a series of operated patients, that removal of the arachnoiditis may lead to some neurological recovery at the level of the injury and below it; this was confirmed in all the patients in this study [23,25]. Falci [24], discussing surgical treatment of posttraumatic cystic and tethered spinal cords, described a group of patients operated for untethering, with one year follow up. The results achieved were: light touch – 2.38 points, pinprick – 3.88 points and motor score – 1.47 points. They were good results at the time, but completely different from the new alternative surgical procedure described here. Imaging techniques such as MRI and CT, which are available for the study of SCI patients, are insufficient to reveal the complete area of the medullar injury and especially the relationship established between the arachnoid, the pia and the dura after the accident and its subsequent consolidation. It is not possible to see either the fibrotic adhesions or the cysts that have formed, and it is often necessary to carry out a conventional myelographic tomography to achieve a more accurate diagnosis [26]. However, complete medullar release at the level of the injury, rostral, and caudal to it permits the extensive arachnoiditis existing in many patients to be removed and precludes the appearance of more attachments. The operation described has some advantages, because the responsible factors for obstructing the shunts and stents are eliminated. It is important to mention that completeness or incompleteness of injuries, the time between the acute traumatic event and this procedure, or the level and type of injury, were not used as selection criteria for this operation. In short, it is also important to note that this procedure has proved safe and without immediate or delayed adverse effects in 100% of the patients.

## Conclusion

This surgical alternative, with complete removal of the arachnoiditis at the level of the injury and of the altered arachnoid on at least two rostral and caudal levels, allows the CSF to circulate as normally as possible, avoiding the relapses that are the reasons for many further surgical operations.

Myelotomies and the use of materials such as shunts and stents are avoided. In the three patients with syringomyelia, the symptoms disappeared post surgery and the control MRI scans showed regression of the cysts after one year and three years. In this particular pathology, with a follow up average of 56 months, there were no repeated relapses.

In all patients who in the T2 MRI scans showed signs of intra medullar hyper intensity, these images disappeared during the first year, and medullar expansion was visible at the end of the third year.

Any medullar atrophy makes this surgical procedure easier, and in no case were there temporary neurological deficits or other post-operative or delayed neurological deterioration.

Finally, a particularly significant conclusion is that all the patients with different levels and types of injuries showed motor and sensory recovery, with greatly improved qualities of life.

## Competing interests

The author(s) declare that they have no competing interests.

**Figure 1:**
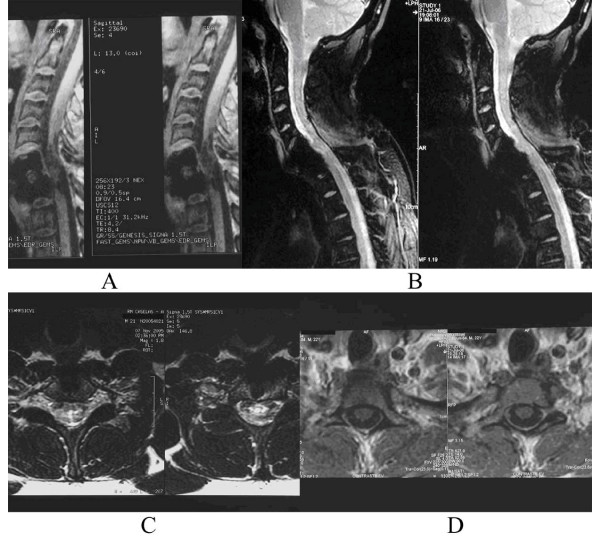
a, b: Tethered cord before and after surgery with a decreased sagittal size of lesion. c, d: Axial view before and after surgery of the same patient. It was shown a better definition of the walls of the spinal cord and without any sub-dural and extra-medullar lesions.

**Figure 2:**
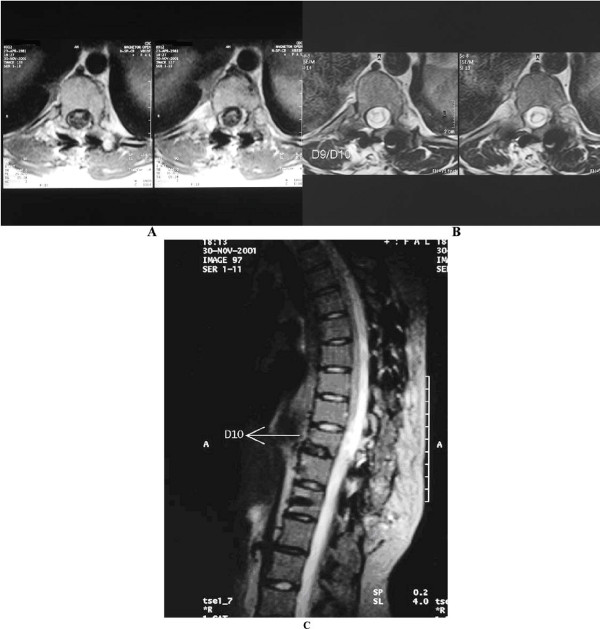
a, b, c: A T-10 tethered cord before (a) and after (b) surgery that shows a cord expansion in the axial pictures.

**Figure 3:**
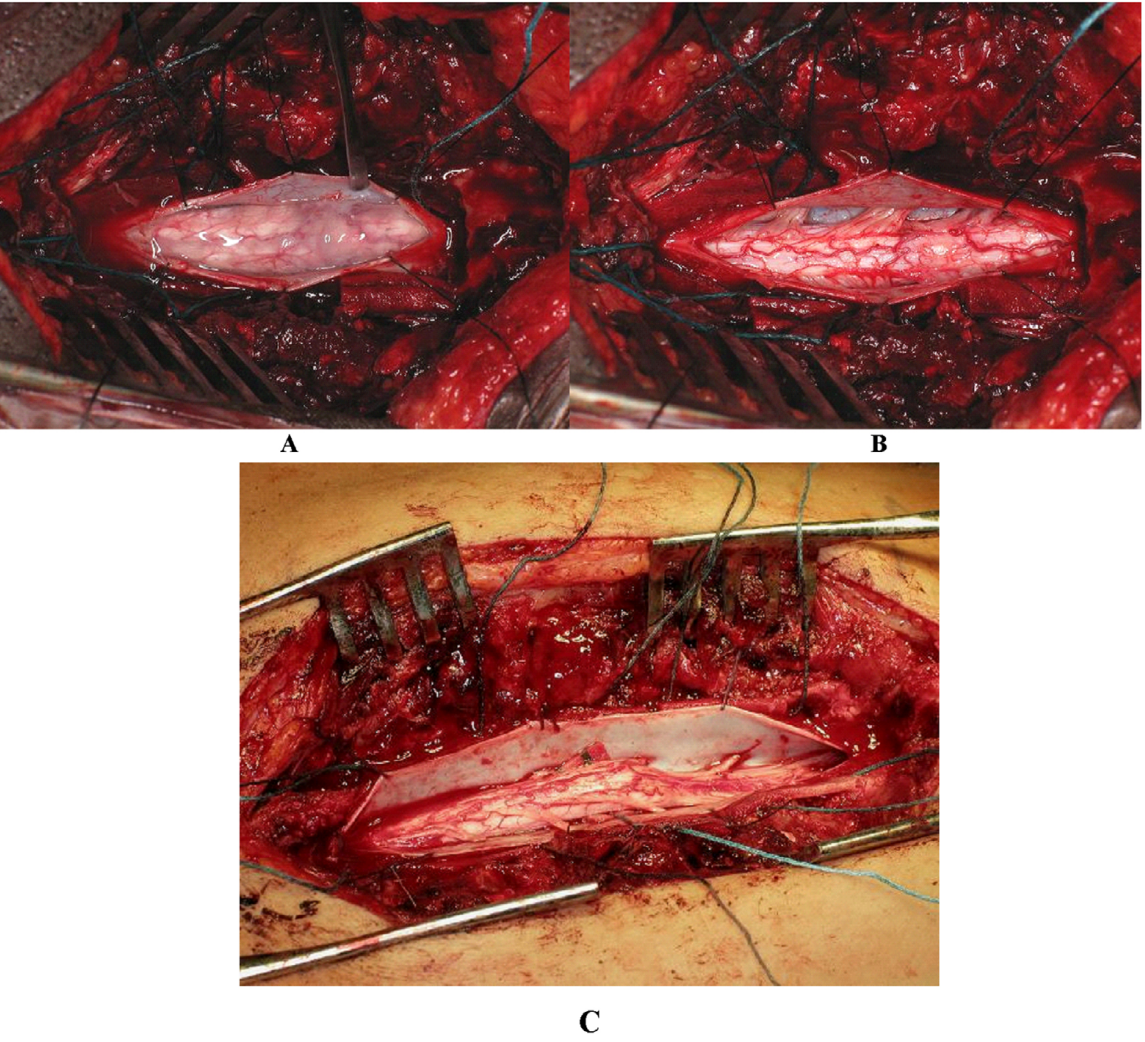
a, b Arachnoid cysts and arachnoiditis before surgery and after complete adhesiolysis. c: Cotton Pattie could be useful to ensure complete anterior adhesiolysis.

## Pre-publication history

The pre-publication history for this paper can be accessed here:



## Supplementary Material

Additional File 1 Wilcoxon signed ranks test. Statistical results of all patients. Click here for file

Additional File 2 Percentage of neurological recovery. Click here for file
