# Supplementary material for: New surgical approach for late complications from spinal cord injury
Source: BMC Surg. 2006 Oct 23;6:12. doi: 10.1186/1471-2482-6-12 (PMC1626077; doi:10.1186/1471-2482-6-12)
Supplement: Additional File 1 — Wilcoxon signed ranks test. Statistical results of all patients. [file 1471-2482-6-12-S1.doc]

# Additional file-1

# MOTOR

# NPar Tests

**Wilcoxon Signed Ranks Test**

Conclusion: There is a statistically significant difference between the final and the initial results in Motor (p-value <0,001).

**MOTOR (FINAL – INITIAL)**

**Descriptive Statistics**

|  | N | Mean | Std. Deviation | Minimum | Maximum | Percentiles | | |
| --- | --- | --- | --- | --- | --- | --- | --- | --- |
|  |  |  |  |  |  | 25th | 50th (Median) | 75th |
| Motor (Initial-Final) | 20 | 20,55 | 5,605 | 12 | 32 | 16 | 20,0 | 25,0 |

The observed mean difference in the results is 20,55 with a standard deviation of 5,605. All patients had positive results, between 12 and 32. 25% of the patients had a difference in the results between 12 and 16. 50% of the patients had a result between 20 and 32. The 25% best succeeded patients had results between 25 and 32.

## TOUCH

### NPar Tests

**Wilcoxon Signed Ranks Test**

Conclusion: There is a statistically significant difference between the final and the initial results in Touch (p-value <0,001).

**TOUCH (FINAL – INITIAL)**

**Descriptive Statistics**

|  | N | Mean | Std. Deviation | Minimum | Maximum | Percentiles | | |
| --- | --- | --- | --- | --- | --- | --- | --- | --- |
|  |  |  |  |  |  | 25th | 50th (Median) | 75th |
| Touch (FINAL-INITIAL) | 20 | 17,5 | 11,87 | 2 | 44 | 8,25 | 14,5 | 23,75 |

The observed mean difference in the results is 17,5 with a standard deviation of 11,87. All patients had positive results, between 2 and 44. 25% of the patients had a difference in the results between 2 and 8,25. 50% of the patients had a result between 14,5 and 44. The 25% best succeeded patients had results between 23,75 and 44.

## PINPRICK

**NPar Tests**

**Wilcoxon Signed Ranks Test**

Conclusion: There is a statistically significant difference between the final and the initial results in Pinprick (p-value <0,001).

**PINPRICK (FINAL – INITIAL)**

**Descriptive Statistics**

|  | N | Mean | Std. Deviation | Minimum | Maximum | Percentiles | | |
| --- | --- | --- | --- | --- | --- | --- | --- | --- |
|  |  |  |  |  |  | 25th | 50th (Median) | 75th |
| Pinprick (FINAL-INITIAL) | 20 | 16,10 | 11,738 | 0 | 41 | 8 | 13 | 20,25 |

The observed mean difference in the results is 16,1 with a standard deviation of 11,738. All patients but one, had positive results, between 2 and 41. 25% of the patients had a difference in the results less or equal than 8. 50% of the patients had a result between 13 and 41. The 25% best succeeded patients had results between 20,25 and 41. Three patients are potential outliers for the substantial higher difference between final and initial pinprick (38; 40; 41).
